# Prognostic classification based on P/F and PEEP in invasively ventilated ICU patients with hypoxemia—insights from the MARS study

**DOI:** 10.1186/s40635-020-00334-y

**Published:** 2020-12-18

**Authors:** Fabienne D. Simonis, Laura R. A. Schouten, Olaf L. Cremer, David S. Y. Ong, Gabriele Amoruso, Gilda Cinella, Marcus J. Schultz, Lieuwe D. Bos, for the MARS consortium, F. M. de Beer, L. D. Bos, G. J. Glas, J. Horn, A. J. Hoogendijk, R. T. van Hooijdonk, M. A. Huson, T. van der Poll, B. Scicluna, L. R. Schouten, M. J. Schultz, M. Straat, L. A. van Vught, L. Wieske, M. A. Wiewel, E. Witteveen, M. J. Bonten, O. L. Cremer, J. F. Frencken, K. van de Groep, P. M. Klein Klouwenberg, M. E. Koster-Brouwer, D. S. Ong, D. M. Verboom

**Affiliations:** 1 Department of Intensive Care, Amsterdam University Medical Centers, location AMC, University of Amsterdam, Meibergdreef 9, 1105 AZ Amsterdam, The Netherlands; 2 Department of Intensive Care Medicine, University Medical Center Utrecht, Utrecht, The Netherlands; 3 Department of Medical Microbiology, University Medical Center Utrecht, Utrecht, The Netherlands; 4 Department of Anesthesia and Intensive Care, University of Foggia, Foggia, Italy; 5 Laboratory of Experimental Intensive Care & Anesthesiology (L·E·I·C·A), Amsterdam University Medical Centers, University of Amsterdam, Amsterdam, The Netherlands; 6 Mahidol–Oxford Tropical Medicine Research Unit (MORU), Mahidol University, Bangkok, Thailand; 7 Nuffield Department of Medicine, University of Oxford, Oxford, UK; 8 Department of Pulmonology, Amsterdam University Medical Centers, University of Amsterdam, Amsterdam, The Netherlands

**Keywords:** Intensive care unit, Invasive ventilation, Hypoxemia, Prognostication, Mortality, Ventilator-free days, ICU-free days, P/F, PaO_2_/FiO_2_, Positive end–expiratory pressure, PEEP

## Abstract

**Background:**

Outcome prediction in patients with acute respiratory distress syndrome (ARDS) greatly improves when patients are reclassified based on predefined arterial oxygen partial pressure to fractional inspired oxygen ratios (PaO_2_/FiO_2_) and positive end–expiratory pressure (PEEP) cutoffs 24 h after the initial ARDS diagnosis. The aim of this study was to test whether outcome prediction improves when patients are reclassified based on predefined PaO_2_/FiO_2_ and PEEP cutoffs 24 h after development of mild hypoxemia while not having ARDS.

**Methods:**

Post hoc analysis of a large prospective, multicenter, observational study that ran in the ICUs of two academic hospitals in the Netherlands between January 2011 and December 2013. Patients were classified into four groups using predefined cutoffs for PaO_2_/FiO_2_ (250 mmHg) and PEEP (5 cm H_2_O), both at onset of hypoxemia and after 24 h: PaO_2_/FiO_2_ ≥ 250 mmHg and PEEP < 6 cm H_2_O (group I), PaO_2_/FiO_2_ ≥ 250 mmHg and PEEP ≥ 6 cm H_2_O (group II), PaO_2_/FiO_2_ < 250 mmHg and PEEP < 6 cm H_2_O (group III), and PaO_2_/FiO_2_ < 250 mmHg and PEEP ≥ 6 cm H_2_O (group IV), to look for trend association with all-cause in-hospital mortality, the primary outcome. Secondary outcome were ICU- and 90-day mortality, and the number of ventilator-free days or ICU-free days and alive at day 28.

**Results:**

The analysis included 689 consecutive patients. All-cause in-hospital mortality was 35%. There was minimal variation in mortality between the four groups at onset of hypoxemia (33, 36, 38, and 34% in groups I to IV, respectively; *P* = 0.65). Reclassification after 24 h resulted in a strong trend with increasing mortality from group I to group IV (31, 31, 37, and 48% in groups I to IV, respectively; *P* < 0.01). Similar trends were found for the secondary endpoints.

**Conclusions:**

Reclassification using PaO_2_/FiO_2_ and PEEP cutoffs after 24 h improved classification for outcome in invasively ventilated ICU patients with hypoxemia not explained by ARDS, compared to classification at onset of hypoxemia.

**Trial registration:**

ClinicalTrials.gov identifier: NCT01905033. Registered on July 11, 2013. Retrospectively registered.

## Background

Hypoxemia commonly develops in invasively ventilated intensive care unit (ICU) patients. While hypoxemia could reflect the presence of pulmonary injury, including acute respiratory distress syndrome (ARDS) [1], it is more likely to be caused by positive pressure ventilation-induced effects on pulmonary circulation [2, 3] or atelectases [4, 5].

Two investigations showed that outcome prediction in adult patients with ARDS greatly improves when patients are reclassified based on predefined arterial oxygen partial pressure to fractional inspired oxygen ratios (PaO_2_/FiO_2_) and positive end–expiratory pressures (PEEP) cutoffs 24 h after the initial ARDS diagnosis [6, 7]. Similar results came from a study in children with ARDS [8]. These findings, at least in part are in line with the recent finding that patients with “resolved” ARDS, i.e., patients who no longer meet the criteria for ARDS after 24 h have a much lower mortality than those with “confirmed” ARDS, i.e., those patients who remain to fulfil these criteria [9].

Whether reclassification based on PaO_2_/FiO_2_ and PEEP cutoffs is also helpful in identifying subsets of ICU patients who develop hypoxemia after start of invasive ventilation is uncertain. The present study, therefore, tested the hypothesis that reclassification after 24 h based on predefined PaO_2_/FiO_2_ and PEEP cutoffs improves outcome prediction in invasively ventilated ICU patients with mild hypoxemia from another cause than ARDS. For this analysis, the database of the conveniently sized prospective “Molecular Diagnosis and Risk Stratification of Sepsis” (MARS) study was used [10].

## Methods

### Design

This was a post hoc analysis of the database of the MARS study that included patients admitted to the mixed medical–surgical ICUs of two university hospitals in the Netherlands: the Amsterdam University Medical Centers (AUMC), location Academic Medical Center, Amsterdam, The Netherlands, and the University Medical Center Utrecht (UMCU), Utrecht, The Netherlands.

Inclusion in the MARS study was with an opt-out consent method approved by the Institutional Review Board of the UMCU with ID no. 10-056C on June 16, 2010. The MARS study was registered at clinicaltrials.gov with study identifier NCT01905033.

### Patients

Consecutive adult patients with an anticipated length of stay in ICU of more than 24 h and admitted between January 2011 and December 2013 were eligible for the parent study. Patients were eligible for participation in this post hoc analysis if they (a) developed hypoxemia, defined as having PaO_2_/FiO2 ≥ 200 and < 300 mmHg at a minimum PEEP of 5 cm H_2_O, within the first 24 h of invasive ventilation and (b) receiving invasive ventilation for at least 24 h. Patients were excluded if (a) meeting criteria for ARDS, (b) not having PaO_2_/FiO_2_ after 24 h, hampering reclassification, or (c) when lost to follow up.

### Data collection and definitions

Baseline and demographic variables were collected on ICU admission to calculate disease severity scores. PaO_2_/FiO_2_ and PEEP at onset of hypoxemia and after 24 h were used for classification and reclassification of patients (see below).

Data collectors were trained in capturing reliable and stable data, thus considering only blood gas analysis results, FiO_2_ and PEEP when a patient was deemed clinically stable, as such ignoring temporary hypoxemic events that were related to accidental patient-ventilator disconnections, obstruction of endotracheal tubes by secretions, airway suctioning, or hemodynamic instability.

Data collectors of MARS were also trained in diagnosing ARDS by using the Berlin definition for ARDS [11].

### Cutoffs for PaO_2_/FiO_2_ and PEEP

Patients were classified into four groups, based on pragmatic cutoffs for PaO_2_/FiO_2_ and PEEP. Using these predefined cutoffs, patients could be classified into four groups: PaO_2_/FiO_2_ ≥ 250 mmHg and PEEP < 6 cm H_2_O (group I), PaO_2_/FiO_2_ ≥ 250 mmHg and PEEP ≥ 6 cm H_2_O (group II), PaO_2_/FiO_2_ < 250 mmHg and PEEP < 6 cm H_2_O (group III), and PaO_2_/FiO_2_ < 250 mmHg and PEEP ≥ 6 cm H_2_O (group IV). These cutoffs were chosen before data extraction and thus before start of the analysis, and were based on the most commonly used setting for PEEP in patient without ARDS [12]. For the PaO_2_/FiO_2,_ we made a pragmatic choice for the middle of the range between 200 and 300.

### Endpoints

The primary endpoint was all-cause in-hospital mortality. Secondary endpoints were ICU- and 90-day mortality, the number of ventilator-free days and alive at day 28, defined as the number of days a patient was alive and not ventilated until day 28 after admittance, and the number of ICU-free days and alive at day 28, defined as the number of days a patient was alive and not in the ICU until day 28 after admittance.

### Standard care

Patients received invasive ventilation according to the local guidelines that prescribed the use of lung-protective settings aiming at tidal volumes < 8 ml/kg predicted bodyweight. PEEP and FiO_2_ uptitrations followed a strict protocol in which recruitment maneuvers were to be performed with every rise in PEEP with hypoxemia, and PEEP and FiO_2_ downtitrations with hyperoxemia. PaO_2_ and SpO_2_ targets were 8 to 10 kPa and 92 to 97%, respectively. Patients in controlled modes of ventilation were assessed at least three times per day to determine whether weaning could start, consisting of a switch to pressure support ventilation. If pressure support ventilation was accepted well, this mode was to be used for further mechanical ventilatory support. Extubation was performed at the discretion of attending physicians, based on general extubation criteria (i.e., the patient was responsive and cooperative, had an adequate oxygenation with a maximum FiO_2_ of 0.4, was hemodynamically stable at low doses inotropes and without uncontrolled arrhythmia, and having a normal temperature) [13]. A restrictive fluid balance, targeting normovolemia, was used with a preference for crystalloid over colloid infusions. In case of shock, the SSC guidelines applicable at the time were followed [14]. Sedation scales were used for analgo-sedation with bolus medication in all patients.

### Statistical analysis

Data were expressed as number or percentages, mean ± standard deviation (SD), or median and interquartile range (IQR) where appropriate. Differences between distributions of categorical variables were analyzed with Pearson chi-square test and with *t* test or Kruskal–Wallis test for numerical variables.

As a first step, trend associations with outcome in the four classification groups based on the predefined PaO_2_/FiO_2_ and PEEP cutoffs at onset of hypoxemia were determined. For the second step, patients were reclassified using the same cutoffs, but after 24 h. *P* values for linear trend for dichotomous outcome variables, i.e., all-cause in-hospital-, ICU-, and 90-day mortality, and *P* values for linear regression for continuous outcome variables, i.e., ventilator-free and ICU-free days and alive at day 28, were calculated for the classification groups, with the groups as the independent variable.

In one post hoc analysis, we compared the four groups for the primary outcome. A pairwise comparison was used at onset of hypoxemia and after 24 h, using a contrast matrix predictor approach. For each mutual comparison between groups, odds ratios and 95% confidence intervals were calculated.

A *P* value of < 0.05 was considered statistically significant. All statistical analyses were conducted with R statistics (v.3.2.2) via the R-studio interface.

## Results

### Patients

**Fig. 1:**
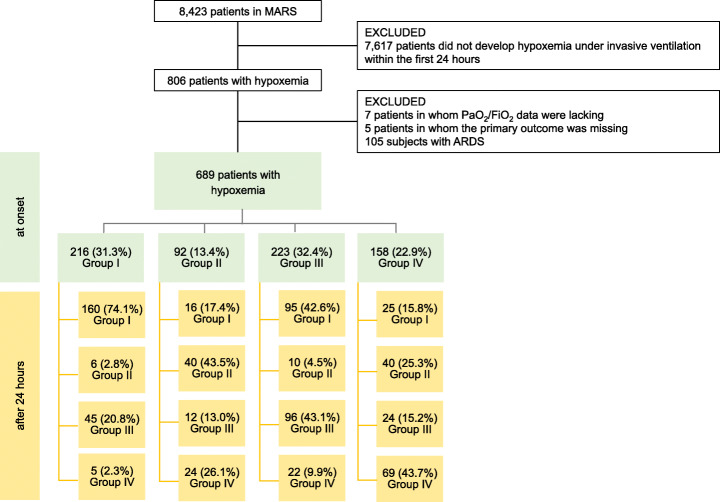
Patient flow and evolution of patient classification; patients were classified at onset of hypoxemia (green boxes) and after 24 h of standard care (yellow boxes); numbers represent the number of patients per group with the fraction of patients

Of 8423 admitted patients in the parent MARS study, 806 patients developed hypoxemia after start of invasive ventilation (Fig. 1). After exclusion of patients who met criteria for ARDS and patients with missing data, 689 patients were left for the current analysis. There were no patients who developed ARDS during follow-up. Overall, all-cause in-hospital mortality was 35% (242/689).

### Evolution of hypoxemia over 24 h

Hypoxemia severity varied significantly from onset till 24 h later **(**Fig. 1). For instance, of 216 patients in group I at onset of hypoxemia, 56 patients (26%) worsened over the next 24 h. Of 92 in group II, 36 patients (39%) worsened. Vice versa, of 223 and 158 patients in group III and IV, 105 patients (47%) and 65 patients (41%) improved.

### Hospital survivors versus non-survivors

Table 1 shows demographics and ventilation characteristics of hospital survivors versus non-survivors. Patients who died in the hospital were older and had higher APACHE IV scores, while patients who survived were more frequently admitted after elective surgery but less frequent after emergency surgery. PaO_2_/FiO_2_, PaO_2_, and PEEP at onset of hypoxemia were not different between hospital survivors and non-survivors, but became different between survivor and non-survivors at 24 h after onset of hypoxemia.

**Table 1:** Baseline characteristics of patients with mild hypoxemia who survived and patients who died in the hospital

Characteristics	Survivors, ***n*** = 447	Non-survivors, ***n*** = 242	***P*** value
APACHE IV score, median [IQR]	71 [56–87]	96 [76–116]	< 0.01
Age, median [IQR]	60 [49–69]	66 [57–75]	< 0.01
Gender, male, *n* (%)	281 (63)	160 (66)	0.40
Admission type, *n* (%)			
Medical	278 (62)	146 (60)	
Elective surgery	63 (14)	22 (9)	0.04
Emergency surgery	106 (24)	74 (31)	
Primary admission specialty, *n* (%)			
Cardiology	67 (15)	34 (14)	
Cardiothoracic surgery	78 (17)	28 (12)	
Ear, nose, and throat	7 (2)	3 (1)	
Gastro-oncologic surgery	7 (2)	5 (2)	
Gastroenterology	7 (2)	4 (8)	
General surgery	83 (19)	42 (17)	
Gynecology and obstetrics	3 (1)	0 (0)	0.03
Hematology	3 (1)	3 (1)	
Head and neck surgery	5 (1)	0 (0)	
Internal medicine	38 (8.5)	13 (5)	
Neurology	64 (14)	57 (24)	
Neurosurgery	45 (10)	35 (14.5)	
Orthopedic surgery	5 (1)	2 (1)	
Pulmonology	28 (6)	11 (4.5)	
Other	2 (0)	4 (2)	
Comorbidities, *n* (%)			
Diabetes mellitus	74 (17)	62 (26)	< 0.01
Immune deficiency	40 (9)	28 (12)	0.30
Cardiovascular disease	143 (32)	76 (31)	0.93
Any malignancy	58 (13)	39 (16)	0.28
COPD	59 (13)	38 (16)	0.43
**Ventilation and other parameters at onset of hypoxemia, median [IQR]**			
Maximum airway pressure, cm H_2_O	19 [16–23]	20 [17–24]	< 0.01
PEEP, cm H_2_O	5 [5–8]	5 [5–8]	0.94
FiO2, %	40 [40–41]	40 [40–41]	0.18
PaO_2_/FiO_2_, mmHg	245 [222–270]	241 [220–273]	0.99
Tidal volume, ml/kg PBW	7.2 [6.2–8.4]	6.9 [6–8.1]	0.19
Respiratory rate, per minute	18 [15–22]	19 [15–24]	0.01
PaO_2_, mmHg	110 [95–129]	105 [93–133]	0.30
PaCO_2_, mmHg	40 [36–46]	38 [34–43]	< 0.01
Dead space fraction	0.1 [0–0.2]	0.1 [0–0.2]	0.07
Use of vasopressors	252 (56)	165 (68)	< 0.01
**Ventilation and other parameters after 24 h, median [IQR]**			
Maximum airway pressure, cm H_2_O	17 [14–21]	18 [15–23]	0.01
PEEP, cm H_2_O	5 [5–6]	5 [5–8]	0.01
FiO2, %	40 [39–41]	40 [39–41]	0.83
PaO_2_/FiO_2_, mmHg	272 [22–324]	248 [213–294]	< 0.01
Tidal volume, ml/kg PBW	7.3 [6.3–8.6]	7.3 [6.4–8.5]	0.88
Respiratory rate, per minute	18 [15–22]	19 [15–25]	0.04
PaO_2_, mmHg	107 [90–133]	101 [88–130]	0.09
PaCO_2_, mmHg	41 [37–46]	39 [34–44]	< 0.01
Dead space fraction	0.1 [0–0.2]	0.1 [0–0.2]	0.04
Use of vasopressors	218 (49)	127 (52)	< 0.01

*APACHE* acute physiology and chronic health evaluation, *COPD* chronic obstructive pulmonary disease, *IQR* interquartile range, *PBW* predicted body weight, *PEEP* positive end–expiratory pressure

### Groups based on the predefined PaO_2_/FiO_2_ and PEEP cutoffs

Demographics and ventilation characteristics of the classification groups based on the predefined PaO_2_/FiO_2_ and PEEP cutoffs at onset of hypoxemia and after 24 h are shown in Table 2. From group I to group IV, FiO_2_ and PaCO_2_ increased; APACHE IV also increased from group I to group IV, but this difference did not reach statistical significance.

**Table 2:** Main characteristics of patients with hypoxemia classified 24 h after onset of hypoxemia

Characteristics	Group I, ***n*** = 296	Group II, ***n*** = 96	Group III, ***n*** = 177	Group IV, ***n*** = 120	***P*** value
APACHE IV score, median [IQR]	76 [59–97]	77 [60–97]	80 [62–98]	85.5 [62–111]	0.06
Age, median [IQR]	62 [49–70]	62 [50–72]	64 [54–72]	62 [53–72]	0.26
Gender, male, *n* (%)	183 (62)	62 (65)	116 (66)	80 (67)	0.74
**Ventilation parameters, median [IQR]**					
Maximum airway pressure, cm H_2_O	16 [13–19]	21 [18–24]	16 [13–20]	22 [19–27]	< 0.01
PEEP, cm H_2_O	5 [5–5]	8 [6–8]	5 [5–5]	8 [7–10]	< 0.01
FiO_2_, %	40 [35–41]	40 [40–41]	40 [40–41]	41 [40–50]	< 0.01
PaO_2_/FiO_2_, mmHg	303 [280–353]	294 [267–339]	212 [190–233]	211 [165–226]	< 0.01
Tidal volume, ml/kg PBW	7.3 [6.4–8.5]	7.3 [6.3–8.4]	7.3 [6.4–8.6]	7.3 [6.5–9]	0.87
Respiratory rate, per minute	17 [14–22]	18 [15–21]	19 [15–23]	18 [15–24]	0.15
PaO_2_, mmHg	120 [105–144]	126 [111–155]	90 [78–98]	94 [82–103]	< 0.01
PaCO_2_, mmHg	39 [35–44]	41 [36–45]	42 [37–48]	41 [36–48]	0.01

*APACHE* acute physiology and chronic health evaluation, *IQR* interquartile range, *PBW* predicted body weight, *PEEP* positive end–expiratory pressure

### Risk of death in the classification groups

The pairwise comparison showed that in-hospital mortality was not significantly different between groups at onset of hypoxemia. Pairwise comparison after 24 h, however, showed a difference in all-cause in-hospital mortality between groups I and IV (OR, 0.48 [95% confidence interval, 0.26–0.86]; *P* value for trend < 0.01) (Table 3). Other differences between groups did not reach statistical significance.

**Table 3:** Intergroup comparisons for hospital mortality at onset of hypoxemia and after 24

Hospital mortality	At onset		After 24 h	
	OR (95% CI)	*P* value	OR (95% CI)	*P* value
Group I vs. II	0.87 (0.43–1.74)	1.00	1.00 (0.51–1.98)	0.97
Group I vs. III	0.79 (0.46–1.34)	1.00	0.73 (0.43–1.24)	0.37
Group I vs. IV	0.97 (0.53–1.74)	1.00	0.48 (0.26–0.86)	< 0.01
Group II vs. III	0.91 (0.46–1.79)	1.00	0.72 (0.35–1.48)	0.48
Group II vs. IV	1.10 (0.53–2.28)	1.00	0.47 (0.22–1.02)	0.05
Group III vs. IV	1.22 (0.68–2.16)	1.00	0.65 (0.34–1.23)	0.32

*OR* odds ratio, *CI* confidence interval

### Secondary outcomes

Overall, all-cause ICU mortality was 25% (169/689) and all-cause 90-day mortality was 42.2% (291/689). The median number of days that patients were free from invasive mechanical ventilatory support and alive at day 28 was 21 [IQR 0–25] days; the median number of days that patients were outside the ICU and alive at day 28 was 22 [IQR 18–25] days. These secondary outcomes were similar in the four classification groups at hypoxemia onset. In line with the change in risk for all-cause in-hospital mortality, reclassification at 24 h resulted in groups with increasing risks for ICU- and 90-day mortality, and decreasing numbers of ventilator-free and ICU-free days and alive at day 28 from groups I to IV (Table 4).

**Table 4:** All-cause in-hospital mortality in groups based on predefined PaO_2_/FiO_2_ and PEEP cutoffs at hypoxemia onset and after 24 h

**At onset**	**Group I,** ***n*** **= 216**	**Group II,** ***n*** **= 92**	**Group III,** ***n*** **= 223**	**Group IV,** ***n*** **= 158**	***P*** **value for trend**
Hospital mortality, *n* (%)	71 (32.9)	33 (35.9)	85 (38.1)	53 (33.5)	0.65
ICU mortality, *n* (%)	50 (23.1)	25 (27.2)	59 (26.5)	35 (22.2)	0.99
90-day mortality, *n* (%)	86 (39.8)	36 (39.1)	103 (46.2)	66 (41.8)	0.40
VFD28, days [IQR]	22 [5–25]	22 [0–24]	21 [0–25]	21 [3–25]	0.28^**#**^
ICUFD28, days [IQR]	23 [19–25]	22 [18–24]	22 [18–25]	22 [17–24]	0.09^**#**^
**At 24 h**	**Group I,** ***n*** **= 296**	**Group II,** ***n*** **= 96**	**Group III,** ***n*** **= 177**	**Group IV,** ***n*** **= 120**	***P*** **value for trend**
Hospital mortality, *n* (%)	90 (30.4)	29 (30.2)	66 (37.3)	57 (47.5)	< 0.01
ICU mortality, *n* (%)	54 (18.2)	19 (19.8)	51 (28.8)	45 (37.5)	< 0.01
90-day mortality, *n* (%)	119 (40.2)	33 (34.4)	75 (42.4)	64 (53.3)	0.03
VFD28, days [IQR]	23 [14–25]	21 [4–24]	22 [0–25]	16 [0–23]	< 0.01^**#**^
ICUFD28, days [IQR]	22 [19–25]	21 [15–25]	22 [19–25]	22 [15–25]	0.02^**#**^

^**#**^A *P* value for linear regression was calculated

*ICU* intensive care unit, *VFD28* ventilator-free days and alive at day 28, *ICUFD28* ICU-free days and live at day 28

## Discussion

The findings of this post hoc analysis can be summarized as follows: (a) classification of invasively ventilated patients at onset of mild hypoxemia using predefined PaO_2_/FiO_2_ and PEEP cutoffs has no predictive value and (b) reclassification 24 h later using the same predefined cutoffs increases the predictive accuracy for clinically important outcomes, and the improvement outcome prediction was found for all five outcomes, i.e., all-cause in-hospital-, ICU-, and 90-day mortality, and the number of ventilator and ICU-free days.

Hypoxemia is a heterogeneous “syndrome” at onset and a second look after 24 h of standard care enables better prognostication. This approach is not new. Indeed, one retrospective analysis of a randomized controlled trial in ARDS patients [15] and one prospective European study in 38 ICUs [16] showed that an improvement in PaO_2_/FiO_2_ within 24 h of standard care is a better predictor of survival than PaO_2_/FiO_2_ at ARDS onset. Similar findings came from studies in pediatric cohorts [8, 17]. The value of reclassification after 24 h compared to stratification at onset was also illustrated by the simple fact that two-third of patients do no longer fulfil the criteria for moderate or severe ARDS at the moment of reassessment [18], and these patients have much better outcomes [19]. The present findings mirror those from two recent investigations in patients with moderate or severe ARDS [6, 7], even though reason for hypoxemia in the present study was very different from these last two studies.

The results of the present study provide additional insight in the predictive value of easy to capture respiratory data captured after 24 h of standard care in invasively ventilated ICU patients that is new for patients suffering from hypoxemia not related to ARDS, patients that have not been studied well so far. If reclassification of patients improves outcome prediction, this would be of value for ICU physicians for determining a patients’ prognosis and setting therapeutic targets, but also to enrich study population by enrolling more homogeneous groups of patients, i.e., with similar prognosis in clinical research.

One salient finding is that the hospital mortality in our cohort was a staggering 35%, which is markedly similar to patients with mild ARDS in the recent “The large observational study to understand the global impact of severe acute respiratory failure” (LUNG SAFE) [1], and also similar to the 31% mortality in patients that had quickly resolving ARDS in a secondary analysis of the same study [9]. Beforehand, we expected patients without ARDS to have lower mortality rates than those with ARDS. However, it should be stated that the present analysis involved a highly specific group. Indeed, patients who were no longer ventilated after 24 h were not included, effectively excluding patients with an easily reversible cause for hypoxemia who were already extubated after 24 h. These are also the patients that have a better expected outcome. Of note, none of the patients in the present cohort developed ARDS. This is in line with another report on a slightly different subgroup captured in the database of the MARS study and also with a recent randomized clinical trial in patients without ARDS but a low PaO_2_/FiO_2_ [20, 21]. Also, more than 50% of the patients ended up in another group when reclassified at 24 h, except for the patients in group I.

Strengths of the present study include its prospective nature of collection and follow-up of data in a large cohort of critically ill patients. A dedicated team of well-trained researchers scored patients daily. This resulted in detailed documentation and validation of ICU events in clinical practice making sure that periods of hypoxemia were not missed, and patients were adequately scored for having ARDS or not. We included a group with a wide variety for reasons for invasive ventilation, increasing external validity. Finally, follow-up was nearly complete.

This study is limited by some specific issues; one important limitation is that patients who were extubated within 24 h or who died within 24 h are not included in this analysis; however, in these patients, classification for outcome is much less relevant. Second, patients who developed hypoxemia after the first day were not included in this study; hence, our findings do not apply for patients with mild hypoxemia due to, e.g., fluid overload during stay in ICU, weaning or any other event that happens later in the course of the admission. Third, some cases of hypoxemia still could have been missed or even misclassified. For instance, the performance of arterial blood gas analyses was not protocolized on predefined time points, although arterial blood gas analyses were performed often, up to every 4 h. Fourth, notwithstanding the specific selection criteria for inclusion, this cohort includes patients with many different underlying etiologies for the hypoxemia, which makes targeting this group with a specific therapy difficult/unlikely. Fifth, as no reliable information on fluid administration was available, we cannot rule out the potential influence of applied fluid regimens on the current findings. Similarly, we lacked data on the etiology of hypoxemia or reason to intubate, and this is a shortcoming for the interpretation. Seventh, our results might be biased to selection for admission to the two tertiary care hospitals in the Netherlands. Any difference in ICU admission practice between countries or institutions might limit the generalizability of the presented results.

## Conclusion

Compared to classification using predefined cutoffs for PaO_2_/FiO_2_ and PEEP at onset of hypoxemia, reclassification after 24 h of standard care using the same cutoffs improves classification for outcome in ICU patients who develop hypoxemia not explained by ARDS.

## Data Availability

The datasets generated and/or analyzed during the current study are available from the corresponding author on reasonable request.

## Abbreviations

APACHE: Acute physiology and chronic health evaluation
ARDS: Acute respiratory distress syndrome
FiO_2_: Fraction of inspired oxygen
ICU: Intensive care unit
IQR: Interquartile range
MARS: “Molecular Diagnosis and Risk Stratification of Sepsis” study
PaO_2_: Arterial oxygen partial pressure
PaO_2_/FiO_2_: Arterial oxygen partial pressure to fractional inspired oxygen ratio
PEEP: Positive end–expiratory pressure
SD: Standard deviation

## References

[1] Bellani G, Laffey JG, Pham T (2016). Epidemiology, patterns of care, and mortality for patients with acute respiratory distress syndrome in intensive care units in 50 countries. JAMA.

[2] Vieillard-Baron A, Matthay M, Teboul JL (2016). Experts’ opinion on management of hemodynamics in ARDS patients: focus on the effects of mechanical ventilation. Intensive Care Med.

[3] Magder S, Guerard B (2012). Heart-lung interactions and pulmonary buffering: lessons from a computational modeling study. Respir Physiol Neurobiol.

[4] Kallet RH, Siobal MS, Alonso JA (2001). Lung collapse during low tidal volume ventilation in acute respiratory distress syndrome. Respir Care.

[5] Cressoni M, Chiumello D, Carlesso E (2014). Compressive forces and computed tomography-derived positive end-expiratory pressure in acute respiratory distress syndrome. Anesthesiology.

[6] Villar J, Fernández RL, Ambrós A (2015). A clinical classification of the acute respiratory distress syndrome for predicting outcome and guiding medical therapy. Crit Care Med.

[7] Bos LD, Cremer OL, Ong DSY (2015). External validation confirms the legitimacy of a new clinical classification of ARDS for predicting outcome. Intensive Care Med.

[8] Khemani RG, Rubin S, Belani S (2015). Pulse oximetry vs. PaO2 metrics in mechanically ventilated children: Berlin definition of ARDS and mortality risk. Intensive Care Med.

[9] Madotto F, Pham T, Bellani G (2018). Resolved versus confirmed ARDS after 24 h: insights from the LUNG SAFE study. Intensive Care Med.

[10] Klouwenberg PMCK, Ong DSY, Bos LDJ (2013). Interobserver agreement of centers for disease control and prevention criteria for classifying infections in critically ill patients. Crit Care Med.

[11] The ARDS Definition Task Force (2012). Acute respiratory distress syndrome: the Berlin definition. JAMA.

[12] Serpa Neto A, Barbas CSV, Simonis FD (2016). Epidemiological characteristics, practice of ventilation, and clinical outcome in patients at risk of acute respiratory distress syndrome in intensive care units from 16 countries (PRoVENT): an international, multicentre, prospective study. Lancet Respir Med.

[13] Determann RM, Royakkers A, Wolthuis EK (2010). Ventilation with lower tidal volumes as compared with conventional tidal volumes for patients without acute lung injury: a preventive randomized controlled trial. Crit Care.

[14] Dellinger RP, Levy MM, Carlet JM (2008). Surviving sepsis campaign: international guidelines for management of severe sepsis and septic shock: 2008. Intensive Care Med.

[15] Bone RC, Maunder R, Slotman G (1989). An early test of survival in patients with the adult respiratory distress syndrome. Chest.

[16] Squara P, Dhainaut JFA, Artigas A, Carlet J (1998). Hemodynamic profile in severe ARDS: results of the European collaborative ARDS study. Intensive Care Med.

[17] Yehya N, Servaes S, Thomas NJ (2015). Characterizing degree of lung injury in pediatric acute respiratory distress syndrome. Crit Care Med.

[18] Villar J, Kacmarek RM, Pérez-Méndez L, Aguirre-Jaime A (2006). A high positive end-expiratory pressure, low tidal volume ventilatory strategy improves outcome in persistent acute respiratory distress syndrome: a randomized, controlled trial. Crit Care Med.

[19] Villar J, Pérez-Méndez L, Kacmarek R (1999). Current definitions of acute lung injury and the acute respiratory distress syndrome do not reflect their true severity and outcome. Intensive Care Med.

[20] Uhel F, Peters-Sengers H, Falahi F et al (2020) Mortality and host response aberrations associated with transient and persistent acute kidney injury in critically ill patients with sepsis: a prospective cohort study. Intensive Care Med. 10.1007/s00134-020-06119-x 10.1007/s00134-020-06119-x PMC7381452 32514599

[21] Simonis FD, Serpa Neto A, Binnekade JM (2018). Effect of a low vs intermediate tidal volume strategy on ventilator-free days in intensive care unit patients without ARDS: a randomized clinical trial. JAMA - J Am Med Assoc.

